# Evaluation of factors related to maternal anxiety during pregnancy among women referred to Tabriz primary care centers

**DOI:** 10.1186/s12888-023-04823-8

**Published:** 2023-05-10

**Authors:** Elmira Mahini, Sevil Hakimi, Hassan Shahrokhi, Behnaz Salahi, Khatereh Olad Baniadam, Fatemeh Ranjbar

**Affiliations:** 1 Medical School, Tabriz University of Medical Sciences, Tabriz, Iran; 2 Research Center of Psychiatry & Behavioral Sciences, Tabriz University of Medical Sciences, Tabriz, Iran; 3 Clinical Research Development Unit, Razi hospital, Tabriz University of Medical Sciences, Tabriz, Iran

**Keywords:** Anxiety, Pregnancy, Prenatal anxiety screening scale (PASS), Risk factors

## Abstract

**Background:**

Maternal anxiety during pregnancy is sometimes considered a normal mechanism to overcome the mother’s mental preoccupation with having a child. However, stress and anxiety might become a medical condition, becoming so severe as to affect the mother’s mental health. Therefore, the present study aimed to investigate factors related to maternal anxiety during pregnancy in women referring to prenatal primary care centers in Tabriz.

**Methods:**

The target population in the present cross-sectional study was the pregnant women referring to primary care centers affiliated with Tabriz University of Medical Sciences in 2018–2019 (n = 533). Sampling was carried out using the random cluster technique (separately for municipal centers). The data were collected using the study tools, including a questionnaire on demographic data, prenatal anxiety screening scale (PASS), and researcher-made questions on maternal anxiety during pregnancy.

**Results:**

In the present study, 37.5% of pregnant women had anxiety. Of all the demographic and background variables, income (P = 0.015), a history of preterm delivery (P = 0.018), and unintended pregnancy (P = 0.022) were significantly related to anxiety. Of the variables of income, a history of preterm delivery, and unintended pregnancy in the regression model, the odds of anxiety were 41% lower in pregnant women with somewhat adequate income than those with inadequate income after correcting for other variables (P = 0.011). In addition, the variable of unintended pregnancy increased the odds of anxiety up to 49% after correcting for other variables (P = 0.023).

**Conclusion:**

The present study showed that income and unintended pregnancy significantly affect maternal anxiety during pregnancy.

**Trial registration:**

The protocol of the study was approved by the Ethics Committee of Tabriz University of Medical Sciences under the code (IR.TBZMED.REC.1398.161).

## Introduction

Pregnancy is an acute period in a woman’s life, during which various emotional, physical, and social changes occur [1]. This period of increased responsibilities might be associated with some emotions such as grief, depression, or anxiety symptoms and signs [2]. Despite scientific advances in physical problems during pregnancy, mental health during pregnancy is still an important subject in the health of pregnant women [3]. Therefore, further studies are necessary to evaluate the symptoms and signs of anxiety and anxiety disorders in pregnant women [4].

Anxiety is among the most common emotional responses in women during the childbearing period, especially during pregnancy [5], including worries, mental preoccupations, fear of pregnancy, delivery, infant health, and future child bringing up [6]. Maternal anxiety during pregnancy is sometimes considered a normal mechanism to combat the mother’s anxiety about becoming a mother and having a child, which might prepare the mother for pregnancy and its associated changes. However, such stress and anxiety turn into a medical condition and become so severe that it might affect the mother’s mental health. Anxiety during pregnancy and after giving birth is associated with some consequences for the mother and child’s health [7]. Severe consequences, including spontaneous abortion, preeclampsia, preterm delivery, and low birth weight, have been reported in such mothers. In addition, anxiety leads to inattention to pregnancy care, inadequate nutrition, and drug abuse and is one of the predicting factors for prenatal depression. Studies have shown that the risk of anxiety during pregnancy is high in women with a history of psychiatric conditions, stressful events [8, 9], traumatic social events, a history of abortion, stillbirth, and preterm delivery [8–11]. Despite the importance of these complications, prenatal anxiety has not been studied sufficiently compared to depression during this period [12, 13]. However, there is evidence that anxiety has a higher prevalence than depression [14]. In a review study in 2016, the prevalence of anxiety disorders was 22.9% during pregnancy and 20% after delivery [15].

The present study aimed to evaluate anxiety-related factors during pregnancy in women referring to primary care centers in Tabriz. In addition, the study was undertaken to determine factors related to anxiety in pregnant women to promote the health of mothers, children, the family, and the community. Proper prenatal screening and therapeutic and educational measures will promote healthcare quality in terms of mental health in pregnant women.

## Materials and methods

### Study aim, design and participants

The target population in the present cross-sectional/descriptive-analytical study was women referring to primary care centers affiliated with Tabriz University of Medical Sciences in 2018–2019, with a minimum gestational age of six weeks. The protocol of the study was approved by the Ethics Committee of Tabriz University of Medical Sciences under the code (IR.TBZMED.REC.1398.161). The sample size was calculated at n = 533 by considering a prevalence of 15% [16], d = 0.03, and a confidence interval of 95% (z = 1.84) based on the Cochrane formula.

$$n=\frac{{{z}_{1}}^{2}{{z}_{2}}^{2}p1 \left(1-{P}^{2}\right)}{{d}^{2}}$$

Sampling was carried out using the random clustering method. To this end, all the primary care centers in Tabriz were numbered, and one-third of these centers were randomly selected using the www.random.org website for sampling. Then the properties of samples were randomly determined in each selected center based on the population data available at each center (the number of pregnant women covered) by considering the total number of participants required (n = 533). The inclusion criteria were pregnancy, referral to the primary care center to receive pregnancy care, having a file at the center, and consent to participate in the study. The exclusion criteria included no interest in participation, lack of time to participate, gestational age of < 6 weeks, low IQ, and cognitive problems.

First, the researcher provided information for the eligible women on the study aims, procedural steps, and the confiodentionality of the collected data. Then the volunteers signed an informed consent form for non-interventional studies.

### Measurements

The data were collected using the study tools, including a questionnaire on demographic data, the prenatal anxiety screening scale (PASS), and researcher-made questions on maternal anxiety during pregnancy. The questionnaires were completed by the subjects with the cooperation of the research team. The PASS questionnaire was designed by Somerville et al. (2013) for screening a wide range of anxiety symptoms and signs, especially in the prenatal period, and its validity and reliability were confirmed. It can be used in different environments, including prenatal clinics, hospitalized patients, and psychiatry centers [17]. This questionnaire was translated into English using the forward-backward method, and its reliability was confirmed by the test-retest method (α = 0.809). In addition, its validity was confirmed by the factor analysis method (0.72) by Ranjbar et al. [18].

This questionnaire surveys mild and severe maternal anxiety during pregnancy and one year after delivery in women > 18 years of age using 31 questions in four constructs: (1) acute anxiety and adaptability (questions 1–10); (2) general anxiety and specific fears (questions 11–18); (3) perfectionism, control, and trauma (questions 19–23); (4) social anxiety (questions 24–31). Each question has four choices with a response score of 0 to 3. To score the questionnaire, the scores of the individual questions are added up, and a score of ≥ 26 is the cut-off point between mild and score anxiety:

0–22: asymptomatic.

21–41: mild anxiety.

42–93: severe anxiety.

Responses to seven item number seven might clinically indicate phobia.

The researcher-made questionnaire was designed based on factors related to maternal anxiety during pregnancy initially by evaluating the literature and systematic reviews in the following domains (1) psychological and psychiatric factors; (2) factors related to social supports and marital relationships; (3) social, demographic, and economic factors; (4) factors related to pregnancy, midwifery, and tragic events. Then the questionnaires were completed by an experienced panel of experts. All the items were extracted based on a literature review and validated by the expert panel. Then the questionnaire was completed by ten patients with valid items as a pilot study.

### Statistical analysis

The data were analyzed with SPSS 21. The normality of data was analyzed with the Kolmogorov-Smirnov test. Frequencies (percentages) and medians (25 and 75 percentiles) were used for qualitative and non-normal quantitative variables, respectively. The qualitative data were analyzed with chi-squared test, and in cases, this test could not be used, Fisher’s exact test was used. Mann-Whitney test was used to analyze quantitative variables in the two non-normal groups. In addition, logistic regression analysis was used to analyze and calculate the effects of quantitative and qualitative data on anxiety. Statistical significance was set at 5%.

## Results

In the present study, 533 pregnant women were evaluated and 37.5% of them had anxiety. Demographic characteristics of pregnant women participating in this study were shown in Table 1. Of all the demographic and background variables of the study, income (P = 0.015), a history of preterm delivery (P = 0.018), and unintended pregnancy (P = 0.022) had a significant relationship with anxiety(Table 2). However, the variables of age, age at marriage, the age difference between the husband and wife, the educational levels of the husband and wife, the occupations of the husband and wife, parity, acute vomiting during pregnancy, a history of stillbirth or infant death, a history of difficult delivery, a history of preeclampsia, a history of gestational diabetes, a family history of OCD, and sleep disorders were not associated with anxiety (P > 0.05).

According to Table 3, of all the variables, of income, a history of preterm delivery, and unintended pregnancy, included in the regression analysis, with the correction for other variables, the risk of anxiety during pregnancy decreased 41% in women with relatively good income than those with insufficient income (P = 0.011). In addition, by correcting for other variables, unintended pregnancy increased the risk of anxiety up to 49%, which was significant (P = 0.023). On the other hand, there was no significant relationship between anxiety and other variables.

**Table 1:** The demographic and background characteristics of the subjects

Characteristics	
**Age, n (%)**	
**<20 years**	37 (6.9)
**21–30 years**	285 (53.5)
**31–40 years**	202 (37.9)
**≥41 years**	9 (1.7)
**Marriage age, n (%)**	
**<20 years**	243 (45.6)
**21–30 years**	266 (49.9)
**31–40 years**	24 (4.5)
**Age difference of the couple, n (%)**	
**Same age**	46 (8.6)
**<5 years**	252 (47.3)
**6–10 years**	193 (36.2)
**≥11 years**	42 (7.9)
**Education, n (%)**	
**Some high school education**	133 (25.0)
**High school graduate**	228 (42.8)
**University**	172 (32.2)
**Education of husband, n (%)**	
**Some high school education**	147 (27.6)
**High school graduate**	218 (40.9)
**University**	168 (31.5)
**Job, n (%)**	
**Housewife**	481 (90.2)
**Employed**	52 (9.8)
**Job of husband, n (%)**	
**Unemployed**	6 (1.1)
**Worker**	82 (15.4)
**Employee**	75 (14.1)
**Other**	370 (69.4)
**Adequacy of monthly income, n (%)**	
**Sufficient**	15 (2.8)
**Somewhat Sufficient**	381 (71.8)
**Insufficient**	135 (25.4)
**Parity, n (%)**	
**First pregnancy**	194 (36.4)
**Not the first pregnancy**	339 (63.6)
**Severe pregnancy vomiting, n (%)**	39 (7.3)
**History of fetal or infant death, n (%)**	140 (26.3)
**History of preterm delivery, n (%)**	47 (8.8)
**History of difficult delivery, n (%)**	15 (2.8)
**History of preeclampsia, n (%)**	2 (0.4)
**History of gestational diabetes, n (%)**	39 (7.3)
**Unintended pregnancy, n (%)**	110 (20.6)
**Family history of OCD, n (%)**	4 (0.8)
**Sleep disorders, n (%)**	21 (3.9)

**Table 2:** Relationship between the demographic and background characteristics of the subjects with anxiety

	Anxiety		
characteristics	Yes	No	P-value
**Age, n (%)**			0.659^*^
**<20 years**	10 (27.0)	27 (73.0)	
**21–30 years**	100 (35.1)	185 (64.9)	
**31–40 years**	74 (36.6)	128 (63.4)	
**≥41 years**	4 (44.4)	5 (55.6)	
**Marriage age, n (%)**			0.473^*^
**<20 years**	90 (37.0)	153 (63.0)	
**21–30 years**	92 (34.6)	174 (65.4)	
**31–40 years**	6 (25.0)	18 (75.0)	
**Age difference of the couple, n (%)**			0.128^*^
**Same age**	20 (43.5)	26 (56.5)	
**<5 years**	79 (31.3)	173 (68.7)	
**6–10 years**	77(39.9)	116 (60.1)	
**≥11 years**	12 (28.6)	30 (71.4)	
**Education, n (%)**			0.070^*^
**Some high school education**	14 (31.1)	31 (68.9)	
**High school graduate**	93 (40.8)	135 (59.2)	
**University**	53 (30.8)	119 (69.2)	
**Education of husband, n (%)**			0.733^*^
**Some high school education**	49 (33.3)	98 (66.7)	
**High school graduate**	81 (37.2)	137 (62.8)	
**University**	58 (34.5)	110 (65.5)	
**Job, n (%)**			0.103^*^
**Housewife**	175 (36.4)	306 (63.6)	
**Employed**	13 (25.0)	39 (75.0)	
**Husband’s job, n (%)**			0.281^**^
**Unemployed**	3 (50.0)	3 (50.0)	
**Worker**	23 (28.0)	59 (72.0)	
**Employee**	31 (41.3)	44 (58.7)	
**Other**	131 (35.4)	239 (64.6)	
**Income, n (%)**			0.015^*^
**Sufficient**	4 (26.7)	11 (73.3)	
**Somewhat Sufficient**	121 (31.8)	260 (68.2)	
**Insufficient**	61 (45.2)	74 (54.8)	
**Gestational age (week)**	37.0 (30.0–38.0)	37.0 (29.3–38.0)	0.544^***^
**Parity, n (%)**			0.501^*^
**First pregnancy**	72 (37.1)	122 (62.9)	
**Not the first pregnancy**	116 (34.2)	223 (65.8)	
**Severe pregnancy vomiting, n (%)**	19 (48.7)	20 (51.3)	0.068^*^
**History of fetal or infant death, n (%)**	50 (35.7)	90 (64.3)	0.899^*^
**History of preterm delivery, n (%)**	24 (51.1)	23 (48.9)	0.018^*^
**History of dystocia, n (%)**	6 (40.0)	9 (60.0)	0.697^*^
**History of preeclampsia, n (%)**	2 (100.0)	0 (0.0)	0.124^**^
**History of gestational diabetes, n (%)**	18 (46.2)	21 (53.8)	0.140^*^
**Uintended pregnancy, n (%)**	49 (44.5)	61 (55.5)	0.022^*^
**Family history of OCD, n (%)**	1 (25.0)	3 (75.0)	0.999^**^
**Sleep disorders, n (%)**	10 (47.6)	11 (52.4)	0.227^*^

*P-value by chi-squared test

**P-value by Fisher’s exact test

***P-value by Mann-Whitney U test

**Table 3:** Logistic regression results of the presence of anxiety in the subgroups of significant variables in the subjects

			95% confidence interval for odds ratio		
Characteristics	Regression coefficient (B)	Odds ratio	Lower	Upper	P-value
**Income, n (%)**					
**Sufficient**	0.717	0.488	0.147	1.620	0.241
**Somewhat Sufficient**	0.527	0.591	0.393	0.888	0.011
**Insufficient (reference group)**	…	…	…	…	…
**History of preterm delivery**	0.704	2.023	1.100	3.721	0.023
**Unintended pregnancy**	0.397	1.487	0.960	2.305	0.076

## Discussion

The present study aimed to evaluate factors related to maternal anxiety during pregnancy in women referring to primary care centers in Tabriz. According to the results, 37.5% of pregnant women had anxiety. A longitudinal study was carried out by Azizi et al. on 75 pregnant women referring to the maternity clinics in hospitals in Bandar-e-Abbas for routine pregnancy care. The data were collected through the Spielberger anxiety questionnaire at three intervals of 29–32, 33–36, and 37–42 weeks. Demographic data were collected, too. The results showed that almost half of the mothers had higher-than-average obvious (42.6%) and latent (45.3%) anxiety scores [19]. Mehraban et al. carried out a cohort study in Ardabil, in which 241 uniparous women with a gestational age of 28–32 weeks were evaluated. The mothers had no known physical and mental conditions, no history of preterm delivery, no midwifery complications, and drug use. The subjects were selected using the multi-step sampling method. The data were collected with the Spielberg anxiety questionnaire and analyzed. The mean obvious and latent anxiety scores of pregnant women with a history of preterm delivery were 14.26 ± 8.8 and 12.27 ± 8.6, respectively. In other words, 20.7% of the subjects had obvious anxiety, and 18.2% had latent anxiety at a mild level [20].

Rezaeian et al. evaluated 176 pregnant women with a gestational age of 24–26 weeks in a descriptive correlational study. The pregnant women were at risk of preterm delivery and had been referred to healthcare centers affiliated with Mashhad University of Medical Sciences. The eligible subjects were selected based on Holbrook’s preterm delivery screening questionnaire. The self-care behaviors were evaluated based on Hart’s pregnancy care questionnaire, and anxiety, depression, and stress were evaluated based on the DASS21 stress, anxiety, and depression questionnaire. In that study, the mean anxiety scores were 8.50 ± 6.5, and 50.6% of the subjects had mild to very severe anxiety [21]. In addition, in a meta-analysis by Fawcett et al., articles that had reported the prevalence of one to eight prevalent anxiety disorders in pregnant women or postpartum women were included in the study. Overall, 2613 records were retrieved, and finally, 26 studies met the inclusion criteria. It was concluded that the prevalence of at least one anxiety disorder or more than one disorder was 20.7% (16.7–25.4%) [22].

In a meta-analysis, Dennis et al. evaluated 23,468 article abstracts, retrieved 783 articles, and 102 studies included 221,974 women from 34 countries. The prevalence of self-reported anxiety symptoms during the first trimester was 18.2% (13.6–22.8%), with 19.1% (15.9–22.4%) and 24.6% (21.2–28.0%) in the second and third trimesters, respectively. The overall incidence of clinical diagnosis for each anxiety disorder was 15.2% (9.0–21.4%) and 4.1% (19–6.2%) for one general anxiety disorder [15].

In a study by Ferreira et al. on 207 pregnant women, the prevalence of anxiety state was 58.5%, and the prevalence of anxiety trait was 53.2% [23]. Silva et al. evaluated 209 pregnant women in the south Millas Gris, Brazil; 62.8% of pregnant women had anxiety, which was more common in the third trimester of pregnancy (42.9%) [24]. Waqas et al. evaluated 500 pregnant women in the obstetrics wards of hospitals in Pakistan. The women were interviewed with a three-section questionnaire. The anxiety levels in the participants were classified as follows: normal (145 women, 29%), borderline (110 women, 22%), and anxious (49%, 245) [10]. In addition, in previous studies, the prevalence of pregnancy-related anxiety in Sweden [25], Bangladesh [26], Pakistan [27], Brazil (obvious anxiety) and (latent anxiety) [28], Iran in Babol (obvious anxiety) [29], Iran in Sari (obvious anxiety) and latent anxiety [30], and in Iran in Qom (latent anxiety) and obvious anxiety [31] were 22%, 29%, 20.4%, 59.5%, and 45.3%, 26.6%, 33% and 44%, and 40.4% and 32.7%, respectively.

Considering the findings on the prevalence of maternal anxiety during pregnancy, the present study is consistent with studies by Bazrafshan et al. and Alipour et al.; however, it is different from other studies that have reported higher or lower prevalence rates. Several reasons might explain the discrepancies between the results of the present study and other studies, including the differences in the tools used to determine anxiety and the study environment. In addition, these discrepancies might be attributed to differences in samples sizes and exclusion and inclusion criteria, such as the disease severity and background factors. In the present study, of all the demographic and background variables evaluated, income (P = 0.015), a history of preterm delivery (P = 0.018), and unintended pregnancy (P = 0.022) were significantly related to anxiety. However, the variables of age, age at marriage, the age difference between the husband and wife, the educational levels of the husband and wife, the occupation of the husband and wife, parity, the history of multiparity, acute vomiting during pregnancy, a history of stillbirth or infant death, a history of a difficult birth, a history of preeclampsia, a history of gestational diabetes, a family history of OCD, a family history of alcohol use, sleep disorders, and a history of receiving oxytocin in a previous pregnancy were not associated with maternal anxiety (P > 0.05). Finally, regression analyses showed that income and unintended pregnancy significantly affected maternal anxiety during pregnancy.

In the study by Mehraban et al., the mean scores of obvious and latent anxiety of pregnant women in their second pregnancy were 13.65 ± 8.2 and 11.75 ± 8.31, respectively. The incidence of preterm delivery increased 2.28 folds with an increase in anxiety scores [20]. In a study by Silva et al., occupation (P = 0.04), the complications of the previous pregnancy (P = 0.00), a history of the risk of abortion in preterm delivery (P = 0.05), the mother’s interest in becoming pregnant (P = 0.01), the number of abortions (P = 0.02), the number of cigarettes smoked daily (P = 0.00), and drug use were related with the incidence of anxiety significantly [24].

In a systematic review by Biaggi et al., 97 articles were selected and analyzed. The results showed that the most important factors related to depression or anxiety before delivery were a lack of a spouse or social support, a history of abuse or domestic violence, a personal history of psychological disorders, unintended pregnancy, traumatic life events, and high perceivable stress, the complications of the current and past pregnancy, a history of abortion or the risk of preterm delivery, and loss of pregnancy [2]. In a meta-analysis by Grigoriadis et al., 1458 article abstracts were evaluated, 306 articles were retrieved, and 29 articles met the inclusion criteria. The anxiety before delivery was associated with an increased risk of preterm delivery (1.39–1.70; OR = 1.54), spontaneous preterm delivery (1.13–1.75; OR = 1.41), mean low birth weight (mean difference= -55.96 gr; -18.31 to -93.62 gr), increased risk of low birth weight (1.48–2.18; OR = 1.80), lower gestational age (mean difference= -0.13 weeks; -0.04 to -0.22 weeks), increased odds of low gestational age (1.26–1.74, OR = 1.48), and lower head circumference (mean difference= -0.25 cm, -0.06 to -0.45 cm) [32].

In addition, in a meta-analysis by Rose et al., of 37 eligible studies, 31 were included in the meta-analysis. They showed that premature birth was significantly associated with anxiety before delivery (OR = 1.46) [33]. In another meta-analysis by Dig et al., 12 studies reported the data of 17,304 women with preterm delivery, and six studies reported the data of 4948 women with low birth weight. Maternal anxiety during pregnancy was significantly associated with the risk of preterm delivery (1.33–1.70, RR = 1.50) and low birth weight (1.32–2.33, RR = 1.76) [34]. However, Yonkers et al. used Edingbrough postpartum depression scale to evaluate pregnant women, reporting that the subscale scores of depression were not significantly correlated with preterm delivery [35]. Considering the results on the relationship between preterm delivery and anxiety, the results of the present study are consistent with all the studies reporting a significant relationship between these two variables, except for the study by Yonkers et al., who reported no significant relationship between preterm delivery and anxiety during pregnancy.

Azizi et al. reported no significant relationship between age, mother’s educational level, occupational status, the number of pregnancies, the type of pregnancy, the baby’s gender on the one hand and the obvious and latent anxiety during the third trimester of pregnancy [19]. Martini et al. carried out a longitudinal prospective study to evaluate maternal anxiety in terms of the newborns’ growth. A total of 306 pregnant mothers were included from outpatient clinics in Germany and evaluated from the early gestational age up to 16 months after delivery. The risk of anxiety during pregnancy in subjects with unintended pregnancy was 1.13 folds higher; however, the difference was not significant statistically (P = 0.5473). In addition, parity increased the risk of anxiety during pregnancy 1.09 times; however, the difference was not significant (P = 0.723) [36]. In the study by Waqas et al., the deductive analysis showed that higher anxiety scores in pregnant women were significantly related to low social support scores, living in rural areas, a history of physical abuse, abortion, C-section, and unintended pregnancy (P < 0.05) [10]. In the study by Ferreria et al., logistic regression analysis showed that the variables of income, educational level, parity, unintended pregnancy, complications in late pregnancy, and a history of smoking and alcohol use were not significantly related to anxiety during pregnancy [23]. The present study is consistent with studies by Biaggi et al. and Waqas et al. concerning the significant relationship between unintended pregnancy and maternal anxiety during pregnancy; however, it is different from studies by Martini et al. and Ferreria et al.

On the other hand, the studies by Ferriroa et al. and Mortini et al. are consistent with the present study since parity was not significantly related to anxiety during pregnancy. Concerning income, the present study is different from the study by Ferreira et al., who reported no significant relationship between income and anxiety during pregnancy. Such a discrepancy might be attributed to differences in income categorization, the tools used to evaluate anxiety, and the study environment.

## Conclusion

The present study showed that income and unintended pregnancy significantly affected maternal anxiety during pregnancy.

## Data Availability

The datasets used and/or analyzed during the current study are available from the corresponding author on reasonable request.

## List of Abbreviations

PASS: Prenatal anxiety screening scale
